# Comparative Assessment of Reverse Osmosis and Nanofiltration for Wine Partial Dealcoholization: Effects on Membrane Performance, Fouling, and Phenolic Compounds

**DOI:** 10.3390/membranes16010048

**Published:** 2026-01-22

**Authors:** Josip Ćurko, Marin Matošić, Karin Kovačević Ganić, Marko Belavić, Vlado Crnek, Pierre-Louis Teissedre, Natka Ćurko

**Affiliations:** 1 Faculty of Food Technology and Biotechnology, University of Zagreb, Pierottijeva 6, 10000 Zagreb, Croatia; marin.matosic@pbf.unizg.hr (M.M.); karin.kovacevic.ganic@pbf.unizg.hr (K.K.G.); marko.belavic@pbf.unizg.hr (M.B.); vlado.crnek@pbf.unizg.hr (V.C.); natka.curko@pbf.unizg.hr (N.Ć.); 2 Unité Mixte de Recherche Œnologie 1366, INRAE, Bordeaux INP, Institut des Sciences de la Vigne et du Vin, Université de Bordeaux, CS 50008-210, Chemin de Leysotte, 33882 Villenave-d’Ornon, France; pierre-louis.teissedre@u-bordeaux.fr

**Keywords:** wine dealcoholization, reverse osmosis, nanofiltration, membrane fouling, phenolic compounds

## Abstract

This study evaluates the partial dealcoholization of red wine using reverse osmosis (ACM3) and nanofiltration (TS80) membranes at 25 and 35 bar, targeting 2% and 4% ethanol reductions. Membrane performance was assessed through fouling analysis and ethanol partitioning, while wine phenolic (flavan-3-ols, anthocyanins) and color characteristics (CIELab parameters) were determined. The 2% reduction process with ACM3 at 25 bar resulted in minimal phenolic changes. The 4% reduction process revealed distinct performance profiles: ACM3 exhibited exceptional stability (3.35–5.30% permeability loss, linear flux decline with R^2^ > 0.93) and ethanol rejection of 17.6–25.5%, while TS80 achieved processing rates three to six times faster with moderate fouling (16.3% loss, 7.7–13.3% rejection). Decreases in flavan-3-ols and anthocyanin concentrations correlated with fouling intensity rather than processing duration. Proanthocyanidin structure remained stable, and color shifts reflected changes in polymeric pigments rather than anthocyanin loss. Reverse osmosis at low transmembrane pressure proved most suitable for quality preservation. The operational trade-off is clear: TS80 offers three to six times faster processing but with greater phenolic loss, while ACM3 requires longer batch times with minimal fouling. Both processes demonstrate that membrane-based dealcoholization without fluid replacement is feasible, providing winemakers with a valuable method to reduce alcohol while preserving quality.

## 1. Introduction

Climate change is one of the most significant challenges facing the wine industry. Rising temperatures and erratic weather patterns have dramatically altered grape composition at harvest, leading to substantially higher ethanol levels in wines. Over the past four decades, average alcohol content in wines has increased by approximately 1% per decade, resulting in a cumulative rise of 3–4% vol. since the 1980s. Temperatures in major European wine-growing regions are projected to increase by a further 2 °C over the next 25 years [1]. This trend is especially pronounced in warm climate zones and Mediterranean regions, where the physiological response of grapevines to heat stress has accelerated phenology and shifted ripening to warmer periods, increasing sugar accumulation and reducing organic acids [2].

Elevated ethanol content in modern wines creates complex problems. High alcohol concentrations negatively affect sensory properties, reducing balance and harmony, shifting aroma perception from fruity to herbaceous notes, and intensifying “hot” or “burning” sensations. Technologically, excess ethanol stresses yeast fermentation, causing sluggish or stuck fermentations and reducing efficiency. Economically, consumer preferences increasingly favor wines with moderate alcohol content, with low-alcohol and no-alcohol wines projected to grow by over 9% annually through 2026 [3]. Regulation (EU) 2019/934 recognizes both membrane processes—including reverse osmosis (RO), nanofiltration (NF), and osmotic distillation (OD)—and thermal methods (vacuum distillation and spinning cone column) as acceptable enological practices for alcohol adjustment while maintaining wine quality and safety [4].

Reverse osmosis has become the most widely adopted dealcoholization technology at an industrial scale, offering low energy consumption and operation at mild temperatures that preserve heat-sensitive compounds. However, the process’s effectiveness and impact on wine composition vary considerably depending on membrane type, transmembrane pressure, and wine matrix composition. Previous studies show that RO can preserve wine quality during modest (2%) dealcoholization, particularly phenolic compounds, but results at higher ethanol reductions (4–5%) remain controversial, with significant variability across grape varieties and wine matrices. Nanofiltration, with its larger pore structure and broader molecular weight cutoff, offers faster processing but typically exhibits higher membrane fouling from phenolic compound accumulation, raising questions about the quality-productivity trade-off. The sensory effects of partial dealcoholization have been documented, with studies showing variable effects depending on grape variety and treatment intensity [5].

Beyond operational performance, the chemical robustness of membrane materials is a critical factor for long-term industrial viability. Thin-film composite (TFC) membranes used in RO and NF applications commonly employ polyamide-based selective layers, whose chemical stability can be compromised under extreme acidic or alkaline conditions, especially during prolonged exposure or repeated cleaning. In strongly acidic environments, hydrolysis of amide linkages may cause chain scission and loss of membrane integrity, while strongly alkaline conditions can promote nucleophilic attack on carbonyl groups, disrupting the polymer backbone and compromising separation performance. These considerations are relevant for wine processing, which involves an acidic matrix and may require repeated cleaning protocols in industrial operation. Recent advances in membrane chemistry indicate that polyurea-based TFC membranes can offer superior chemical durability, maintaining high rejection performance (>91%) after 45 days of continuous exposure to extreme acidic or alkaline conditions [6]. While the present study uses commercially available polyamide-based membranes operated under conditions recommended by the manufacturer, acknowledging these material dependencies and emerging solutions provides important context for evaluating membrane durability in wine dealcoholization applications.

This study evaluates the partial dealcoholization of red wine using batch membrane processes without external water addition as an innovative method to ensure regulatory compliance while maintaining compositional integrity. The research investigates reverse osmosis (ACM3) and nanofiltration (TS80) membranes under different transmembrane pressures (25 and 35 bar) and target alcohol reductions (2% and 4%) using Plavac mali wine with an initial ethanol content of 14.96% *v*/*v*. Membrane performance is characterized through fouling analysis, ethanol partitioning, and flux decline mechanisms, with a comprehensive assessment of impacts on wine composition, including phenolic and chromatic characteristics. The main objectives are to define optimal membrane operating parameters that balance processing efficiency with wine quality preservation and to demonstrate that membrane-assisted dealcoholization without fluid replacement is a technically feasible solution to climate-induced alcohol elevation in wine.

## 2. Materials and Methods

### 2.1. Membrane Specifications and Filtration System

Two commercial spiral-wound thin-film composite membranes were used for partial ethanol removal: the ACM3 membrane for reverse osmosis (RO) and the TS80 membrane for nanofiltration (NF) (Mann + Hummel, Ludwigsburg, Germany). Both membranes had an effective area of 0.23 m^2^ (size 1812), a maximum operating pressure of 41 bar, and a maximum operating temperature of 45 °C. The ACM3 RO membrane has a NaCl rejection of 99.3% with a molecular weight cutoff of less than 0.1 kDa, while the TS80 NF membrane has a MgSO_4_ rejection of 99.2% with a molecular weight cutoff of 0.5–1 kDa. According to manufacturer specifications, both membranes are polyamide thin-film composite (TFC) spiral-wound elements with a cleaning pH tolerance of 1.0–12.0 and a free chlorine tolerance of less than 0.1 ppm. Filtration experiments were conducted using the native wine matrix (pH = 3.76 ± 0.02; Table 1) without pH adjustment. Oxidizing disinfectants, such as free chlorine, were avoided to prevent potential oxidative damage to the polyamide selective layer.

Dealcoholization trials were performed on a benchtop cross/tangential flow filtration system (Sterlitech, Auburn, WA, USA) equipped with integrated pressure monitoring, permeate collection, and temperature control. Both membranes were tested at transmembrane pressures (TMP) of 25 and 35 bar to achieve target ethanol reductions of 2% and 4% by volume, with all trials performed in duplicate. The initial wine volumes were 3.375 L for 2% reduction and 4.375 L for 4% reduction. Wine temperature was maintained at 20 °C using a refrigerated recirculating chiller (Newington, NH, USA) with a heat exchanger in the feed line. The feed tank was blanketed with inert gas (nitrogen or argon) to prevent wine oxidation during filtration.

### 2.2. Batch Dealcoholization Process

Research was conducted using Plavac mali (Pelješac, Croatia) wine from the 2024 vintage with 14.96% vol. ethanol (control wine). General physicochemical characteristics of the control wine are presented in Table 1. Before pressurized filtration, the wine was recirculated through the membrane system without applied pressure until the target temperature (20 °C) was reached. Once thermal equilibrium was achieved, a constant transmembrane pressure was applied. Permeate was collected in defined volumes: 790 mL for a 2% ethanol reduction target and 1580 mL for a 4% reduction. Operational parameters– permeate volume, feed and concentrate pressures, concentrate flow, and wine temperature—were recorded at regular intervals. Batch durations were measured from the start of pressurization to the final permeate collection.

The initial wine volumes (3.375 L for 2% reduction; 4.375 L for 4% reduction) and corresponding permeate collection targets (790 mL and 1580 mL, respectively) were calculated based on ethanol mass balance to achieve absolute ethanol reductions of 2% or 4% (*v*/*v*) from the initial wine ethanol content of 14.96% (*v*/*v*). These volumes accounted for expected ethanol partitioning between permeate and retentate, which varies depending on membrane rejection characteristics. After vacuum evaporation removed ethanol from the collected permeate, the ethanol-depleted permeate fraction was quantitatively returned to the retentate, yielding final dealcoholized wine volumes equal to the initial volumes with no external fluid additions, in compliance with EU Regulation 2019/934. Final ethanol concentrations were analyzed according to the OIV method [7], with all compositional data, including achieved ethanol reductions, reported in Table 1.

The collected permeate was then subjected to mild vacuum evaporation using a rotary evaporator (EV400, LabTech, Hopkinton, MA, USA) connected to a vacuum pump (VP18 PLUS, LabTech, Hopkinton, MA, USA) at 30 °C to selectively remove ethanol. The ethanol-depleted permeate was subsequently returned to the retentate, yielding the final dealcoholized wine. This batch reconstitution approach ensured the final product contained only original wine fractions, with no added water or fluids, thus maintaining compliance with EU regulations [4].

### 2.3. Membrane Performance Characterization

The permeate flux (*J*) was calculated using the following formula:

(1) $$J\;=\;V_{p}/(A\times \;t)$$

where *V_p_* is the permeate volume (L), *A* is the membrane surface area (m^2^), *t* is the process duration (h), and *J* (L/m^2^ h) is the permeate flux.

To calculate the ethanol retention factor (*R*_e_), the following formula was used:

(2) $$R_{e}=1\;-(C_{p}/C_{f})$$

where *C_e_* is the ethanol concentration in the permeate, and *C_f_* is the ethanol concentration in the feed tank.

The volume reduction factor (*VRF*) was calculated as follows:

(3) $$VRF\;=\;V_{f}\;/\;V_{r}$$

where *V_f_* is the initial wine volume (L), and *V_r_* is the retentate volume (L).

Before and after each dealcoholization trial, membrane clean water permeability (CWP) was measured at four transmembrane pressures (5, 10, 15, and 20 bar). For each pressure, 100 mL of deionized water was collected, and flux was calculated using the equation above. Each measurement is the average of five consecutive determinations. Clean water permeability was then calculated as follows:

(4) CWP = J / TMP

where *J* (L/m^2^·h) is the permeate flux, *TMP* is the transmembrane pressure (bar), and *CWP* is the membrane’s clean-water permeability (L/m^2^·h·bar). *CWP* loss was expressed as the percentage change from initial values, providing a quantitative assessment of irreversible fouling and membrane degradation during wine processing.

### 2.4. Physicochemical and Spectrophotometric Characterization of Wine

Basic enological physicochemical parameters were analyzed according to the OIV methods [7]. Spectrophotometric analyses were conducted using UV-VIS spectrophotometer (Specord 50 PLUS, Analytik Jena, Jena, Germany) as follows: (i) total phenolics (TP) by using the Folin–Ciocalteu method, expressed in mg gallic acid equivalents (GAE)/L [8]; (ii) total tannins (TT) by the acid hydrolysis method, expersed in g/L [9]; (iii) total anthocynins by the bisulfite bleaching method, expressed in mg/L [10] and (iv) chromatic characteristics (L*, a*, b*, C*_ab_ and h values) using the CIELab [7].

### 2.5. UHPLC-FLUO-MS Analysis of Monomeric and Oligomeric Flavan-3-Ols

Monomeric and oligomeric flavan-3-ols [(+)-catechin (CAT), (−)-epicatechin (EC), procyanidin dimers B1, B2, B3, B4 and trimers C1 and T2] were analyzed on a UHPLC-Fluo-MS system (1290 Infinity II LC, Agilent Technologies, Santa Clara, CA, USA) according to the method earlier described [11]. The separation was performed on LiChrospher RP-18 (250 mm × 4 mm, 5 µm) column (Merck, Darmstadt, Germany) with the mobile phases consisting of (i) solvent A, water/formic acid (99.5:0.5, *v*/*v*), and (ii) solvent B, acetonitrile/formic acid (99.5:0.5, *v*/*v*). Detection and identification of flavan-3-ols was conducted as previously proposed [12]. The concentrations of all individual flava-3-ols, as well as their sums [Sum_mon_ (sum of catechin and epicatechin), Sum_dim_ (sum of procyanidin dimers B1, B2, B3, and B4), and Sum_trim_ (sum of procyanidin trimers C1 and T2)], were obtained as proposed earlier and expressed in mg/L [11].

### 2.6. Structural Characterization of Proanthocyanidins by UHPLC–DAD–QQQ–MS

Proanthocynidin structural characteristics, mean degree of polymerization (mDP), percentage of galloylation (%G), and percentage of prodephinidines (%P) were determined by the method of phloroglucinolysis [13]. Analysis was conducted on a UHPLC-DAD-QQQ-MS system (1290 Infinity II LC, Agilent Technologies, Santa Clara, CA, USA) equipped with both DAD and QQQ-MS (6460 Triple Quadrupole mass spectrometer) with a heated electrospray ionization probe connected in series via the DAD cell outlet. The separation was performed on a Kinetex PFP column (150 mm × 3 mm, 2.6 μm) (Phenomenex, Le Pecq Cedex, France) with the mobile phases consisting of (i) solvent A, water/formic acid (99.9:0.1, *v*/*v*), and (ii) solvent B, methanol/formic acid (99.9:0.1, *v*/*v*), with a flow rate set at 400 μL/min. The elution gradient conditions were as follows: 2% B isocratic from 0 to 2 min, 2–20% B linear from 2 to 5 min, 20–40% B linear from 5 to 25 min, 40–60% B linear from 25 to 38 min, 60–98% B linear from 38 to 40 min, 98% B isocratic from 40 to 45 min, and 98–2% B linear from 45 to 46 min, with re-equilibration of the column from 46 to 48 min under initial gradient conditions. ESI parameters were operating in negative mode, where gas temperature and flow were 350 °C and 5 L/min, respectively; sheath gas temperature and flow were 250 °C and 10 L/min, respectively; capillary voltage was 4500 V and the cell accelerator voltage was 8 V. All data was processed using MassHunter Qualitative Analysis software version B.05 (Agilent Technologies, Santa Clara, CA, USA). The reaction cleavage products were determined as proposed earlier [11,14].

### 2.7. UHPLC-DAD Analysis of Anthocyanins

Five anthocyanin-3-*O*-glucosides (-3-*O*-glucosides of delphinidin, cyanidin, petunidin, peonidin, and malvidin) and four major acylated anthocyanins (-3-*O*-(6-*O*-acetyl)glucoside of peonidin and malvidin-and -3-*O*-(6-*O*-acetyl)glucoside of peonidin and malvidin) were determined on a UHPLC-Fluo-MS system (1290 Infinity II LC, Agilent Technologies, Santa Clara, CA, USA) as previously described [11]. The separation was performed on a Nucleosil C18 (250 mm × 4.6 mm, 5 µm) column (Phenomenex, Torrance, CA, USA) with the mobile phases consisting of (i) solvent A, water/formic acid (95:5, *v*/*v*), and (ii) solvent B, acetonitrile/formic acid (95:5, *v*/*v*). Detection and identification were performed at 520. The concentrations of all anthocyanins, as well as their sums [AcyGlc (sum of anthocyanin glucosides), AcyAc (sum of anthocyanin acetylglucosides), and AcyCm (sum of anthocyanin coumaroylglucosides)] were obtained as proposed earlier [11] using an external standard calibration curve of malvidin-3-*O*-glucoside chloride, and expressed in mg/L.

### 2.8. Statistical Analysis

Membrane dealcoholization experiments were performed in duplicate (*n* = 2 independent batches), and each wine sample was analyzed in triplicate (*n* = 3 analytical replicates per batch). Data are presented as mean ± standard deviation. The statistical data analysis was carried out using the Analysis of Variance (ANOVA) by Statistica V.7 software (Statsoft Inc., Tulsa, OK, USA). Tukey’s HSD test was used as a comparison test when samples were significantly different after ANOVA (*p* < 0.05) for chemical analyses.

## 3. Results and Discussion

### 3.1. Membrane Filtration Performance

Membrane-based dealcoholization of wine has traditionally relied on blending treated permeate with the original wine or blending concentrated retentate with RO/NF permeate, approaches that El Rayess et al. [15] and Kumar et al. [16] have reviewed as standard in the field. Both approaches may require adding water to the retentate to control osmotic pressure, as Ivić et al. [17] and Gonçalves et al. [18] showed for RO systems, where the volume loss caused by permeation is routinely compensated by water addition to the wine retentate. Such water addition conflicts with premium winemaking regulations, where El Rayess et al. [15] note that the compositional integrity of wine must be strictly maintained. To address this, we developed a batch process without external water addition: (i) RO or NF membrane treatment fractionates wine into ethanol-enriched permeate and quality-critical retentate; (ii) the permeate undergoes mild vacuum evaporation to yield ethanol-free or low-ethanol permeate; (iii) this treated permeate is returned to the retentate to achieve the target ethanol reduction. This strategy ensures that the final product consists entirely of original wine fractions with no extraneous additions, making it inherently regulation-compliant. For mild dealcoholization (2% and 4% absolute ethanol reduction), this approach avoids extreme ethanol removal demands on membrane or thermal steps, thereby preserving volatile aromatics and heat-sensitive quality attributes. In this process context, membrane performance was evaluated in terms of productivity, fouling behavior, and ethanol partitioning into the permeate.

**Figure 1:**
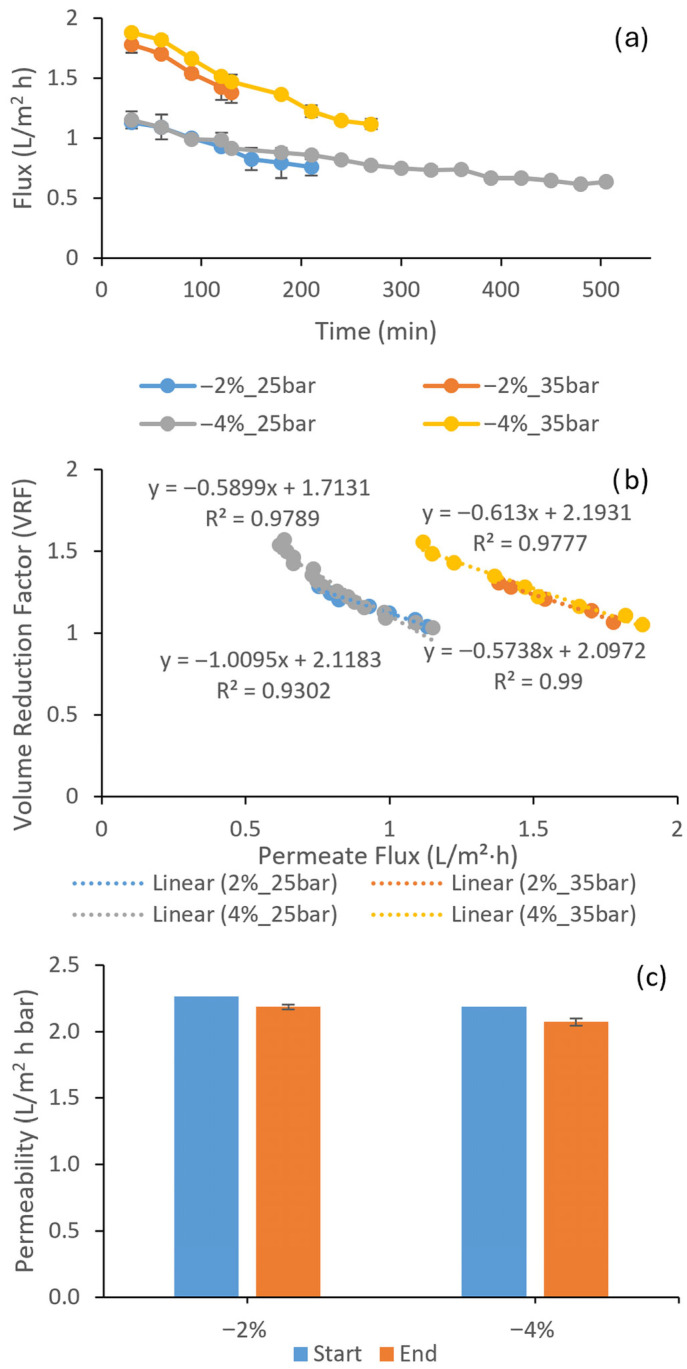
Flux (**a**), VRF–flux relationship (**b**), and clean water permeability (**c**) loss of the ACM3 RO membrane during batch dealcoholization of red wine.

Permeate flux declined almost linearly over time for both membranes under all operating conditions. For the RO membrane ACM3, initial fluxes at 30 min ranged from 1.13 to 1.87 L/m^2^ h (1.13 L/m^2^ h at 2% target, 25 bar; 1.15 L/m^2^ h at 4% target, 25 bar; 1.77 L/m^2^ h at 2% target, 35 bar; 1.87 L/m^2^ h at 4% target, 35 bar), decreasing to final values of 0.82 L/m^2^ h (2% target, 25 bar), 1.38 L/m^2^ h (2% target, 35 bar), 0.63 L/m^2^ h (4% target, 25 bar), and 1.11 L/m^2^ h (4% target, 35 bar) by the end of the runs (Figure 1a). This corresponds to average flux declines of 33%, 22%, 45%, and 41% for the four conditions, respectively. The batch runs lasted 130–150 min (for a final ethanol reduction of 2%) or 270–505 min (for 4% reduction), representing the time needed to collect enough permeate so that, after evaporation and return, the final wine would contain 2% or 4% less ethanol than the original. Higher pressure reduced the collection time.

**Figure 2:**
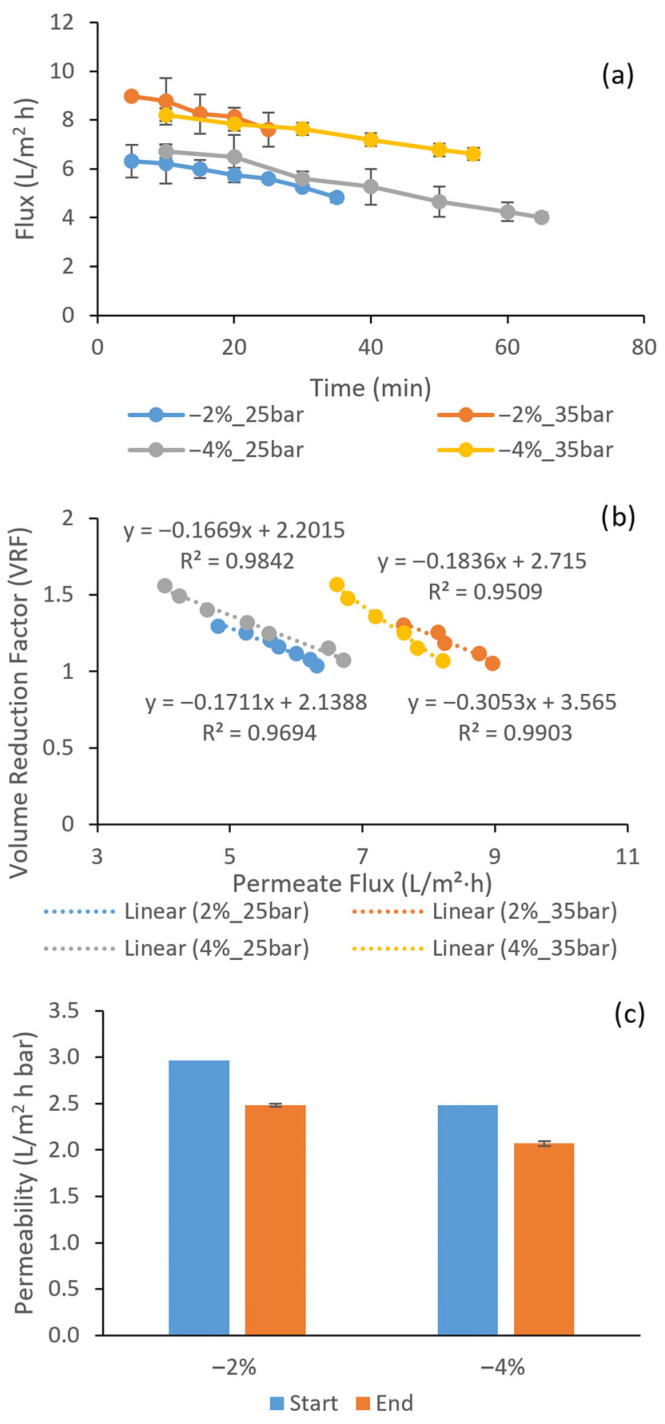
Flux (**a**), VRF–flux relationship (**b**), and clean-water permeability loss (**c**) of the TS80 NF membrane during batch dealcoholization of red wine.

The NF membrane TS80 showed substantially higher initial fluxes at 5–10 min, ranging from 6.3 to 8.96 L/m^2^ h (6.3 L/m^2^ h at 2% target, 25 bar; 6.7 L/m^2^ h at 4% target, 25 bar; 8.96 L/m^2^ h at 2% target, 35 bar; 8.2 L/m^2^ h at 4% target, 35 bar), decreasing to final values of 4.8 L/m^2^ h (2% target, 25 bar), 4 L/m^2^ h (4% target, 25 bar), 7.6 L/m^2^ h (2% target, 35 bar), and 6.6 L/m^2^ h (4% target, 35 bar) by the end of the runs (Figure 2a). This corresponds to average flux declines of 24%, 40%, 15%, and 20% for the four conditions, respectively. The batch runs lasted 25–35 min (for 2% final reduction) or 55–65 min (for 4% reduction), representing the time needed to collect sufficient permeate for reconstitution. Thus, under the tested conditions, TS80 achieved approximately a 3–6 fold reduction in process time compared with ACM3, which is consistent with the higher water permeability and larger molecular weight cutoff typically reported for NF membranes in wine applications [16,17]. Similar trends have been observed at pilot scale, where NF membranes operated at lower transmembrane pressures than RO provided higher permeate fluxes and permeates richer in ethanol (relative to RO), reducing the permeate volume and osmotic pressure difference needed to achieve a given alcohol reduction [18].

The volume reduction factor (VRF)–flux relationships further clarify the mechanisms governing flux decline. For ACM3, VRF–flux plots were strongly linear, with regression coefficients (R^2^) of 0.979, 0.990, 0.930, and 0.982 for the four conditions (2%_25 bar, 2%_35 bar, 4%_25 bar, 4%_35 bar) (Figure 1b), while TS80 showed similarly linear behavior with R^2^ values of 0.969, 0.951, 0.984, and 0.990 (Figure 2b). The slopes ranged from −0.574 to −1.009 for ACM3 and from −0.166 to −0.305 for TS80, reflecting the higher absolute fluxes and partial passage of low molecular weight solutes through the NF membrane. This pronounced linearity indicates that, for both membranes, flux decline was predominantly driven by the progressive increase in osmotic pressure as the retentate was concentrated, rather than by a transition to a strongly fouling-limited or limiting flux regime. A recent investigation on Mavrud wine reported similar linear VRF–flux relationships (R^2^ > 0.94) for both NF and RO membranes under comparable operating conditions, supporting the conclusion that osmotic pressure effects dominated flux decline when fouling was controlled [19]. Previous studies on red wines have often reported more pronounced deviations from linear VRF–flux behavior, attributed to the presence of colloids and macromolecular fouling [17,20]. In this context, the present linear relationships support the conclusion that concentration effects dominated and that irreversible fouling was limited under the chosen conditions.

Clean water permeability measurements before and after the dealcoholization experiments quantify the extent of irreversible fouling. For ACM3, the loss of CWP across all runs was limited to 3.35–5.30%, corresponding to retention of more than 94% of the initial pure water permeability after approximately 2230 min of cumulative operation on a phenolic-rich red (Table 2) wine at 20 °C and 25–35 bar (Figure 1c). Previous studies on RO treatment of red wines typically report fouling indices of about 22–56%, depending on the wine matrix and operating pressure, indicating that the present values are at the very low end of the published range [20,21,22]. In a recent study on Mavrud wine, RO membranes showed CWP losses of 8–12% after comparable cumulative operation times, further highlighting the exceptionally low fouling observed here for ACM3 [19]. The combination of strong linearity in the VRF–flux plots and small CWP loss, therefore, indicates a stable, osmotic pressure-controlled regime with only minor irreversible deposition on ACM3. The stability of the ACM3 system, indicated by minimal CWP loss and linear VRF–flux behavior, contrasts with nanofiltration membranes, which typically experience higher fouling loads due to the accumulation of phenolic compounds on their surfaces and within their pore structures [20,23].

The NF membrane TS80 showed a higher but still moderate CWP loss of 16.26–16.63%, corresponding to approximately 83–84% retention of intrinsic permeability. These values are lower than many literature reports for NF membranes on red wines, where losses of 26–32% are common [20,22]. Recent work on Bulgarian Mavrud wine using NF membranes reported CWP losses of 19–24% under similar operating conditions, confirming that the present TS80 values (16–17%) are at the lower end of the observed range for phenolic-rich red wines [19]. The higher fouling of TS80 compared to ACM3 is consistent with its much shorter cumulative operating time (about 7 h across all parallel runs) combined with higher absolute fluxes, which tend to increase concentration polarization and interactions between phenolic compounds and the membrane surface. The accumulation of phenolic compounds on NF membranes reflects their tendency to adsorb onto membrane polymers through hydrogen bonding and electrostatic interactions, particularly at wine pH (3.1–3.8), where phenolic compounds mostly exist in equilibrium between neutral and negatively charged forms [23]. This pH-dependent behavior explains the preferential fouling of nanofiltration systems compared to reverse osmosis, where tighter membrane pores and higher operating pressures minimize opportunities for such interactions [20,23].

Beyond pore size, membrane structure and surface properties significantly contribute to the observed differences in phenolic retention between ACM3 and TS80. ACM3’s reverse osmosis design (<0.1 kDa MWCO) features tighter molecular packing [16], which limits accessible surface area for phenolic adsorption and restricts penetration of oligomeric compounds into the membrane matrix. In contrast, TS80’s nanofiltration structure (0.5–1 kDa MWCO) offers substantially greater internal surface area and more accessible pore walls, facilitating adsorptive interactions with flavan-3-ol oligomers through hydrogen bonding, π-π stacking, and hydrophobic forces, with surface energy and polarity playing critical roles in these interactions. At wine pH, phenolic compounds mostly exist in equilibrium between neutral and negatively charged forms, influencing their affinity for membrane surfaces and explaining the membrane-dependent retention patterns observed experimentally. This accounts for the membrane-specific phenolic losses documented in Section 3.2: at 4% reduction (25 bar), ACM3 caused 10% dimer losses (from 149.87 to 135.62 mg/L) compared to TS80’s 25% losses (from 149.87 to 112.50 mg/L), despite TS80’s processing time being 3–6 times shorter (55–65 min vs. 270–505 min). The substantially higher CWP loss for TS80 (16.26–16.63%) compared to ACM3 (3.35–5.30%) directly reflects this difference in membrane-phenolic affinity, confirming that membrane structural properties and surface chemistry, not just pore size, determine fouling severity and compound retention in wine dealcoholization. It should be noted that the present conclusions are based on duplicate membrane experiments (*n* = 2 independent batches), which effectively discriminate large performance differences between membranes but provide limited statistical power for resolving modest differences between operating conditions.

**Figure 3:**
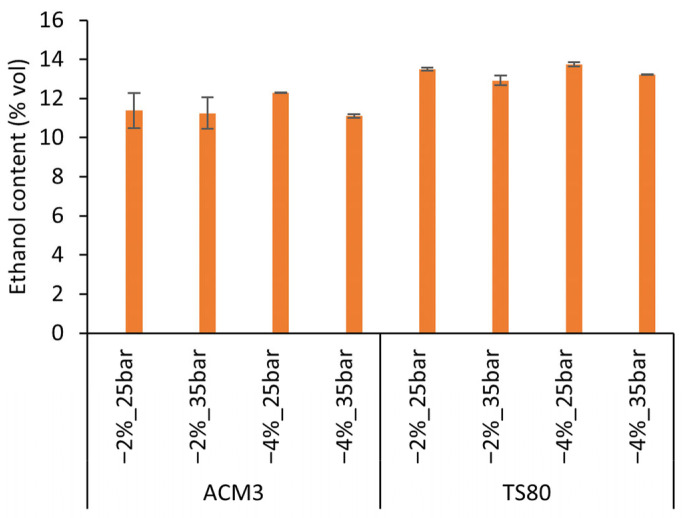
Ethanol concentrations in permeates from ACM3 and TS80 under different operating pressures (25 and 35 bar) and target ethanol reductions (2% and 4%).

Ethanol partitioning into the permeate was evaluated relative to the original wine, which contained 14.96% (*v*/*v*) ethanol (Figure 3). For ACM3, the average permeate ethanol concentrations at the end of batch operation ranged from 11.10% to 12.28% (*v*/*v*), corresponding to ethanol rejections of approximately 17.6–25.5%. For TS80, permeate ethanol concentrations were higher, between 12.92% and 13.75% (*v*/*v*), giving rejection values of about 7.7–13.3%. These rejection values fall within the ranges reported for wine dealcoholization, where RO membranes typically show ethanol rejections of about 8–26% and NF membranes about 5–18% under comparable conditions [20,22]. Work on Mavrud wine using similar NF membranes reported ethanol rejections of 9–15% and RO rejections of 20–28% under concentration-mode operation, closely aligning with the present values for TS80 (7.7–13.3%) and ACM3 (17.6–25.5%) [19]. The ethanol rejection values reflect fundamental differences in membrane selectivity, directly linked to pore structure and separation mechanisms. Reverse osmosis membranes such as ACM3 exhibit higher ethanol rejection (17.6–25.5%) due to their tighter pore structure (<0.1 kDa MWCO), which restricts the passage of small polar molecules through solution–diffusion transport [16], effectively retaining both ethanol and phenolic compounds in the retentate while allowing only water to permeate preferentially. In contrast, nanofiltration membranes such as TS80 have larger pores (0.5–1 kDa MWCO) and demonstrate lower ethanol rejection (7.7–13.3%), allowing greater permeation of ethanol-rich liquid through combined pore-flow and diffusion mechanisms [19,20]. This structural difference has dual consequences: TS80 produces permeates richer in ethanol (12.92–13.75% *v*/*v* vs. 11.10–12.28% *v*/*v* for ACM3), reducing the permeate volume required for target dealcoholization, but simultaneously increases membrane–phenol interactions due to higher flux and larger pore accessibility, as evidenced by the threefold higher irreversible fouling (16.3% CWP loss vs. 3.35–5.30% for ACM3) and correspondingly greater phenolic losses documented in Section 3.2.

Taken together, the flux, fouling, and ethanol data show that RO and NF offer distinct yet complementary performance profiles for batch dealcoholization of wine. The RO membrane ACM3 provides slower processing but exceptionally low irreversible fouling and highly stable, osmotic pressure-controlled flux decline, with ethanol rejection values at the upper end of those reported for wines and only minor permeability loss over extended operation. The NF membrane TS80 delivers much higher fluxes and 3–6 times shorter processing times, with ethanol rejection in the typical NF range and moderate, controllable fouling that remains compatible with standard cleaning protocols. The linear VRF–flux relationships for both membranes and the modest changes in CWP indicate that, under the selected conditions, both processes can be operated in a predictable, controllable regime. This provides a robust membrane basis for the permeate evaporation and return strategy, while the choice between RO and NF can be guided by the desired balance between productivity, fouling control, and retention of wine constituents.

While the present results demonstrate the technical feasibility of batch dealcoholization without external water addition, long-term membrane stability and performance over repeated cycles require careful consideration for industrial implementation. The reported CWP losses—3.35–5.30% for ACM3 after approximately 2230 min of cumulative operation and 16.26–16.63% for TS80 after approximately 420 min—represent irreversible fouling that persists after water rinsing, indicating that effective cleaning protocols are essential for maintaining performance across multiple batch cycles. The effectiveness of such cleaning protocols for removing adsorbed phenolic compounds—particularly polymerized flavan-3-ol aggregates that form strong π-π stacking and hydrogen bonding interactions with membrane polymers—remains uncertain and requires systematic evaluation. Critically, the absence of external water addition in the present process eliminates dilution-induced flux recovery between batches, meaning that membrane regeneration must rely entirely on cleaning effectiveness rather than partial fouling reversal through concentration gradients. This places greater demands on regeneration protocols compared to traditional blending processes, where residual water can partially restore flux. The present study demonstrates single-batch feasibility. However, evaluation of cumulative fouling behavior, cleaning efficiency, or flux recovery over repeated cycles should be further considered for industrial deployment.

### 3.2. Wine Phenolic and Chromatic Characteristics

Overall, the −2% process had minimal impact on the physicochemical characteristics of the wine, with most parameters remaining comparable to those of the control (Table 1). The −2% dealcoholization using the RO (ACM3) membrane at lower TMP (25 bar) showed no significant differences from the control wine across all analyzed parameters (*p* < 0.05), highlighting the ability of this mild process to preserve the wine’s original composition, consistent with earlier findings for low-intensity RO processes [18,22]. Wine pH also remained stable in both the −2% and −4% processes, indicating that dealcoholization did not affect acid-base balance, as similarly reported in other RO/NF wine studies [21]. More pronounced effects became apparent only when ethanol reduction increased to −4%. These changes can be attributed to the absence of fluid replacement after ethanol removal (water, vegetation water, or wine), which produced a concentration effect in the dealcoholized wines, an effect well documented in membrane-based dealcoholization [15]. As a result, wine density was the most affected parameter and was significantly higher in the −4% compared to the −2% wines (*p* < 0.05). A similar trend was observed for lactic acid concentration and total acidity (*p* < 0.05), though differences between the ACM3 and TS80 membranes remained modest under both degrees of ethanol reduction.

Concentrations of total phenolics (TP) were generally not significantly affected by the dealcoholization processes (*p* < 0.05) (Table 2), which is consistent with observations that RO membranes strongly retain phenolic compounds [22]. However, more pronounced differences between the control and dealcoholized wines were observed in total tannin (TT) concentrations (Table 2), where the effects of membrane type appeared to depend on the degree of ethanol reduction. In the −2% processes, the use of ACM3 membranes did not result in significant differences in TT concentrations compared to the control wine (*p* < 0.05), whereas the −2% process using TS80 membranes led to significantly lower TT concentrations, consistent with the lower selectivity of NF membranes for tannins [17]. In contrast, the −4% process resulted in significantly higher total tannin levels compared to the control wine, with no significant differences observed between the ACM3 and TS80 membranes (*p* < 0.05). As previously discussed, this increase is likely associated with the stronger concentration effect caused by the greater ethanol removal during the −4% treatment.

Furthermore, within the −2% process, a trend similar to that observed for total tannins appeared in the concentrations of flavan-3-ol monomers, dimers, and trimers when comparing the TS80 and ACM3 membranes (Table 2). However, unlike total tannins, flavan-3-ol concentrations were strongly influenced not only by membrane type but also by transmembrane pressure. Dealcoholization using the ACM3 membrane at 25 bar showed no significant differences compared to the control wine, whereas applying a higher TMP (35 bar) resulted in significantly lower concentrations of procyanidin B2, as well as in the overall sum of dimers and trimers (*p* < 0.05). In contrast, dealcoholization using the TS80 membrane at both 25 and 35 bar resulted in significantly lower concentrations of all analyzed flavan-3-ols and their overall sums in the wine compared to the control, particularly in the process conducted at higher TMP. In addition, greater losses were observed in the concentrations of procyanidins B1 and B3, as well as in the overall sum of dimers. It is important to note that the differences obtained between ACM3 and TS80 membranes reflect the combined effects of size exclusion and solute–membrane interactions. ACM3’s very tight selective layer (<0.1 kDa MWCO) provides strong steric hindrance, while TS80’s larger nominal MWCO (0.5–1 kDa) should retain oligomeric flavan-3-ols (dimers and trimers) more effectively than monomers, which are closer to the NF cutoff and may partially permeate. Because the permeate was fully returned to the retentate in our closed-loop process, any permeation would not result in net losses in the final wine; thus, the observed concentration decreases are most consistent with fouling-related entrapment and irreversible adsorption rather than solute transfer into a discarded permeate fraction. TS80’s more open structure provides greater accessible internal surface area and pore-wall contact for adsorption (hydrogen bonding, π–π stacking, hydrophobic interactions) [20,23], and its 3–6 times higher flux (6.3–8.96 vs. 1.13–1.87 L·m^−2^·h^−1^) intensifies concentration polarization, locally enriching phenolics and accelerating adsorption. This helps explain why TS80 shows 25–27% dimer losses at 4% reduction (25 bar) versus 10–12% for ACM3, despite shorter processing times, and is consistent with the larger CWP decline for TS80 (16.26–16.63%) compared with ACM3 (3.35–5.30%). In addition, greater losses of oligomeric flavan-3-ols at higher TMP can also be explained by intensified concentration polarization and fouling, which increase membrane–phenolic interactions and promote irreversible adsorption. Higher transmembrane pressure increases the thickness of the concentration polarization layer at the membrane surface, thereby increasing the residence time of phenolic compounds within this enriched microenvironment and promoting irreversible adsorption onto the membrane matrix. This pressure-dependent enhancement of fouling-induced losses has been consistently documented in nanofiltration and reverse osmosis studies on wine [20,23].

Changes in flavan-3-ols during the −4% process also depended on membrane type and TMP (Table 2). Compared with the TS80 membrane, the ACM3 membrane had a more favorable effect on the wine, and processes conducted at lower pressures with both ACM3 and TS80 resulted in smaller losses of flavan-3-ols. The process using the ACM3 membrane at 25 bar resulted in significantly lower concentrations of B1, B2, and the total sum of dimers and trimers (*p* < 0.05). Among the −4% treatments, this condition produced the smallest flavan-3-ol losses, making it the most suitable option for wine dealcoholization at the 4% level. A similar trend was observed at 35 bar, though this treatment resulted in significantly lower concentrations of the aforementioned compounds compared to the 25 bar process (*p* < 0.05). Within the −4% process, the TS80 membrane, as already noted for the −2% process, resulted in significant losses of all determined flavan-3-ols compared to the control. These losses were more pronounced at 35 bar than at 25 bar, due to additional decreases in B1, B3, and C1, as well as in the overall sum of monomers, dimers, and trimers (*p* < 0.05). The greater losses observed with the TS80 nanofiltration membrane compared with RO can be attributed to its looser structure and broader molecular cutoff, which facilitate the formation of a thicker fouling layer enriched in flavan-3-ol aggregates and phenolic complexes [15,23]. The magnitude of quality losses is associated with fouling intensity rather than processing duration. Despite TS80’s processing time being 3 to 6 times shorter (55–65 min vs. ACM3’s 270–505 min for a 4% reduction), its significantly higher fouling load (16.3% CWP loss vs. ACM3’s 3.4–5.3%) resulted in 2.5 times greater losses of proanthocyanidin dimers (27% vs. 10–12%) and total anthocyanins (27% vs. 2–3%). This inverse relationship between exposure time and quality loss demonstrates that membrane–phenol interactions during fouling, rather than cumulative contact time, are the dominant mechanism of compound removal in batch dealcoholization processes.

At both reduction levels, higher transmembrane pressure (35 vs. 25 bar) consistently intensified flavan-3-ol losses, with this effect more pronounced for TS80 than for ACM3. This pressure-dependent behavior aligns with the fouling data (Section 3.1), where increased TMP elevated concentration polarization and accelerated foulant accumulation, thereby increasing the residence time of phenolic compounds at the membrane interface and promoting irreversible adsorption. The differential retention rates observed among phenolic compound classes reflect fundamental differences in molecular properties that govern membrane–compound interactions. Flavan-3-ol oligomers (dimers and trimers) exhibited significantly greater losses than monomers across all treatments, with this effect most pronounced for TS80 at 4% reduction, where dimers decreased by 25% (from 149.87 to 112.50 mg/L), trimers by 19% (from 32.95 to 26.58 mg/L), and monomers by 17% (from 62.83 to 52.41 mg/L) (Table 2). This oligomer-selective loss pattern arises from synergistic molecular factors: (i) higher molecular weight with increasing polymerization degree increases steric accessibility to membrane pores and enhances hydrophobic interactions with the polymer matrix; (ii) increased hydrophobicity promotes stronger adsorption onto membrane surfaces through π-π stacking and van der Waals forces, with surface energy and polarity playing critical roles in governing these interactions [24,25]; and (iii) greater conformational flexibility of dimers relative to larger trimers enables more efficient penetration into the concentration polarization layer and membrane pore structure, explaining why procyanidin dimers B1 and B2 showed the most pronounced losses. Despite the observed quantitative losses of flavan-3-ols due to membrane fouling and adsorption, these processes did not substantially alter the structural composition of proanthocyanidins, as previously reported [22]. Additionally, both dealcoholization processes (−2% and −4%) did not affect the structural characteristics of the wine, as no significant changes were observed in the values of mDP, %G, or %P for either membrane type or pressure (Table 2). The only exception was the TS80 membrane, which showed a slightly lower percentage of %G in the −2% process at both applied pressures. However, this trend was not observed in the −4% process.

While anthocyanins generally exhibit lower membrane affinity than flavan-3-ols due to their ionic character and reduced aggregation tendency, this protective effect was membrane-dependent. ACM3 preserved anthocyanins under all conditions, whereas TS80-4% caused significant losses (25–27% for total anthocyanins, *p* < 0.05), indicating that the thicker fouling layer on TS80 can entrap even highly polar compounds when fouling intensity reaches critical levels (Table 3). Similar stability has been reported in membrane-based wine dealcoholization, where anthocyanins show weaker membrane affinity than flavan-3-ols [26,27]. This resistance stems from anthocyanins existing predominantly as flavylium cations at wine pH, conferring high polarity and hydrophilicity that reduce both membrane surface adsorption and aggregation tendency compared to the neutral hydroxyl-rich structure of flavan-3-ols. The cationic character also creates electrostatic repulsion from negatively charged membrane surfaces at wine pH, further limiting anthocyanin–membrane interactions except under severe fouling conditions, where physical entrapment within the dense fouling layer becomes the dominant retention mechanism, as observed for TS80 at 4% reduction with 16.3% CWP loss. Significant decreases compared with the control wine were observed only in the sum of anthocyanin coumaroylglucosides for both the −2% and −4% processes using the ACM3 membrane, and in cyanidin-3-*O*-glucoside for the −2% process using the TS80 membrane (*p* < 0.05). Furthermore, significantly lower concentrations of total and free anthocyanins (anthocyanin-3-*O*-glucosides and acylated anthocyanins) were detected only in the −4% process using the TS80 membrane (*p* < 0.05). However, no significant differences were observed between processes performed at different pressures, supporting the observation that TMP has minimal influence on anthocyanin retention in RO/NF systems [15,22].

Although the concentrations of free anthocyanins were largely unchanged, changes in the chromatic characteristics of the wine were more pronounced (Table 3). This indicates that wine color was influenced primarily by alterations in polymeric pigments and copigmentation complexes rather than by free anthocyanins alone [26], likely resulting from modifications within the flavan-3-ol fraction. Within the −2% processes, no significant differences were observed between the control wine and the wine dealcoholized using the ACM3 membrane at 25 bar. In contrast, dealcoholization with the ACM3 membrane at 35 bar, as well as with the TS80 membrane at both pressures, caused a significant increase in L*, b*, and h values, accompanied by a significant decrease in a* and C*_ab_ values. These shifts in chromatic parameters indicate a decrease in red color intensity and a shift toward lighter and more yellowish hues. An increase in L* reflects higher lightness, meaning the wine appears less intensely colored. The decrease in a* and C*_ab_ denotes reduced redness and lower chroma, while increases in b* and h indicate a movement from red-purple tones toward more orange–yellow hues. Such changes are consistent with a loss of polymeric pigments and copigmentation interactions, which are major contributors to red wine color stability [26]. In contrast, the −4% process using TS80 coupled with free anthocyanin losses (25–27%) with further color deterioration, indicating that severe fouling impacts both pigment classes: free anthocyanins through direct entrapment, and polymeric pigments through disruption of flavan-3-ol–anthocyanin copigmentation complexes. Within the −4% processes, increasing TMP further enhanced lightness, leading to significantly higher L* values, particularly in wines dealcoholized using the TS80 membrane compared with ACM3. Moreover, wines subjected to −4% dealcoholization showed significantly higher b* and h values than the control, indicating a continued shift toward more yellowish hues. However, unlike the −2% processes, a decrease in a* and C*_ab_ values was not evident, suggesting that the −4% treatments affected hue and lightness more strongly than red saturation.

## 4. Conclusions

This study demonstrates that partial wine dealcoholization using reverse osmosis (ACM3) and nanofiltration (TS80) without fluid replacement is both technically feasible and compliant with regulations. A 2% ethanol reduction with ACM3 at 25 bar caused no significant changes in physicochemical, phenolic, or chromatic characteristics, while a 4% reduction led to greater flavan-3-ol losses due to concentration effects and membrane fouling, though proanthocyanidin structure remained stable. ACM3 showed exceptional stability (3.35–5.30% CWP loss after 2230 min, R^2^ > 0.93 flux linearity, 17.6–25.5% ethanol rejection), whereas TS80 achieved three- to six-fold faster processing (55–65 min vs. 270–505 min for 4% reduction) with moderate fouling (16.3% CWP loss, 7.7–13.3% ethanol rejection). Quality losses correlated with fouling intensity rather than processing duration: TS80’s higher fouling resulted in 2.5 times greater proanthocyanidin losses (27% vs. 10–12%) and anthocyanin losses (27% vs. 2–3%) compared to ACM3, despite substantially shorter exposure times.

The operational trade-off is clear: ACM3 provides superior quality preservation with minimal fouling and is optimal for premium wine production at 2–4% alcohol reduction, whereas TS80 offers significantly faster throughput but with greater phenolic losses, making it suitable for higher-volume commercial applications where processing speed is prioritized. Both membrane processes demonstrated that climate-induced alcohol elevation can be addressed without compromising wine compositional integrity, providing winemakers with a practical and regulation-compliant technological solution.

## Figures and Tables

**Table 1:** Effect of partial dealcoholization membrane process operating parameters on general physicochemical characteristics in Plavac mali wine.

Operating Parameters									
	Control	Dealcoholized Wine							
Membrane	/	ACM3	ACM3	TS80	TS80	ACM3	ACM3	TS80	TS80
TMP (bar)	/	25	35	25	35	25	35	25	35
Process	/	−2%	−2%	−2%	−2%	−4%	−4%	−4%	−4%
Analytical parameters									
Density (g/cm^3^)	0.9956 ^c^	0.9956 ^c^	0.9951 ^d^	0.9948 ^e^	0.9947 ^e^	0.9978 ^a^	0.9977 ^ab^	0.9975 ^b^	0.9975 ^b^
Ethanol (%vol.)	14.96 ^a^	12.97 ^b^	12.93 ^b^	13.00 ^b^	12.99 ^b^	10.96 ^c^	10.95 ^c^	10.96 ^c^	10.97 ^c^
Reducing sugar (g/L)	3.50 ^b^	3.50 ^b^	3.33 ^c^	3.29 ^c^	3.23 ^c^	3.64 ^a^	3.59 ^ab^	3.54 ^ab^	3.52 ^b^
Total acidity (g/L)	5.00 ^b^	5.00 ^b^	5.00 ^b^	5.05 ^b^	5.08 ^b^	5.35 ^a^	5.27 ^a^	5.31 ^a^	5.35 ^a^
Malic acid (g/L)	nd	nd	nd	nd	nd	nd	nd	nd	nd
Lactic acid (g/L)	0.91 ^b^	0.91 ^b^	0.91 ^b^	0.93 ^b^	0.95 ^b^	1.22 ^a^	1.22 ^a^	1.19 ^a^	1.19 ^a^
pH	3.76 ^a^	3.76 ^a^	3.76 ^a^	3.76 ^a^	3.76 ^a^	3.75 ^a^	3.75 ^a^	3.74 ^a^	3.74 ^a^

Data are presented as average values over three analytical replicates (*n* = 3). ANOVA to compare data; different letters in each row indicate statistical difference between wines (Tukey’s test, *p* < 0.05). Abbreviations: nd, not detected. Target ethanol reductions of 2% and 4% (*v*/*v*) from control (14.96% *v*/*v* ethanol) were achieved as shown by final measured ethanol concentrations of 12.93–13.00% and 10.95–10.97% (*v*/*v*), respectively. All analyses were performed on final dealcoholized wines standardized to 1500 mL volume by blending ethanol-depleted permeate with retentate, ensuring comparability across treatments.

**Table 2:** Effect of partial dealcoholization operating parameters on the proanthocyanidins concentrations and structural characteristics in Plavac mali wine.

Operating Parameters									
	Control	Dealcoholized Wine							
Membrane	/	ACM3	ACM3	TS80	TS80	ACM3	ACM3	TS80	TS80
TMP (bar)	/	25	35	25	35	25	35	25	35
Process	/	−2%	−2%	−2%	−2%	−4%	−4%	−4%	−4%
Analytical parameters									
TP *	2930.0 ± 79.6 ^ab^	2935.8 ± 57.7 ^ab^	2901.8 ± 115.3 ^b^	2753.9 ± 111.7 ^b^	2752.4 ± 33.6 ^ab^	2918.2 ± 44.9 ^ab^	3029.5 ± 118.2 ^a^	2904.5 ± 46.4 ^ab^	2934.0 ± 44.8 ^ab^
TT **	5.04 ± 0.10 ^b^	4.94 ± 0.03 ^b^	4.98 ± 0.13 ^b^	4.80 ± 0.07 ^c^	4.88 ± 0.07 ^bc^	5.31 ± 0.03 ^a^	5.31 ± 0. 06 ^a^	5.28 ± 0.06 ^a^	5.29 ± 0.08 ^a^
(+)-C *	46.61 ± 0.32 ^a^	46.31 ± 0.54 ^ab^	45.38 ± 0.32 ^b^	44.19 ± 0.34 ^c^	44.15 ± 0.47 ^c^	46.56 ± 0.46 ^a^	46.27 ± 0.25 ^ab^	39.75 ± 0.61 ^d^	39.10 ± 0.49 ^d^
(−)-EC *	16.22 ± 0.38 ^ab^	16.13 ± 0.33 ^ab^	16.38 ± 0.38 ^a^	15.05 ± 0.19 ^c^	15.64 ± 0.50 ^abc^	15.31 ± 0.59 ^abc^	15.22 ± 0.85 ^bc^	12.66 ± 0.29 ^d^	12.55 ± 0.07 ^d^
B1 *	90.82 ± 0.55 ^a^	90.86 ± 0.57 ^a^	88.48 ± 0.90 ^ab^	87.24 ± 1.31 ^b^	80.12 ± 2.32 ^c^	86.91 ± 0.93 ^b^	83.21 ± 2.69 ^c^	75.95 ± 1.27 ^d^	72.38 ± 1.00 ^e^
B2 *	40.95 ± 1.20 ^a^	41.06 ± 0.91 ^a^	32.87 ± 0.36 ^c^	37.18 ± 0.71 ^b^	36.93 ± 0.58 ^b^	30.90 ± 0.72 ^d^	30.66 ± 0.59 ^d^	21.01 ± 0.80 ^e^	21.23 ± 1.04 ^e^
B3 *	8.07 ± 0.16 ^a^	8.08 ± 0.14 ^a^	8.13 ± 0.23 ^a^	7.22 ± 0.23 ^b^	6.31 ± 0.15 ^c^	7.77 ± 0.15 ^a^	7.74 ± 0.15 ^a^	7.24 ± 0.03 ^b^	6.64 ± 0.08 ^c^
B4 *	10.02 ± 0.08 ^a^	9.99 ± 0.16 ^ab^	9.79 ± 0.14 ^ab^	9.79 ± 0.09 ^ab^	9.69 ± 0.18 ^b^	10.04 ± 0.10 ^a^	9.77 ± 0.08 ^ab^	8.31 ± 0.18 ^c^	8.19 ± 0.17 ^c^
C1 *	11.70 ± 0.04 ^a^	11.60 ± 0.14 ^ab^	11.50 ± 0.10 ^ab^	11.33 ± 0.15 ^b^	10.99 ± 0.06 ^c^	11.38 ± 0.18 ^ab^	11.30 ± 0.13 ^bc^	9.82 ± 0.17 ^d^	9.07 ± 0.21 ^e^
T2 *	21.29 ± 0.22 ^a^	21.28 ± 0.23 ^a^	20.67 ± 0.21 ^ab^	19.87 ± 0.18 ^c^	19.87 ± 0.15 ^c^	21.21 ± 0.20 ^a^	21.10 ± 0.20 ^a^	16.77 ± 0.21 ^d^	16.55 ± 0.13 ^d^
∑ Mon *	62.83 ± 0.41 ^a^	62.44 ± 0.67 ^a^	61.76 ± 0.50 ^a^	59.23 ± 0.43 ^b^	59.79 ± 0.54 ^b^	61.87 ± 0.87 ^a^	61.49 ± 1.08 ^a^	52.41 ± 0.89 ^a^	51.65 ± 0.55 ^c^
∑ Dim *	149.87 ± 1.69 ^a^	149.99 ± 1.26 ^a^	139.27 ± 0.46 ^bc^	141.43 ± 1.32 ^b^	133.04 ± 1.94 ^de^	135.62 ± 0.97 ^cd^	131.38 ± 3.38 ^e^	112.50 ± 1.54 ^f^	108.44 ± 1.50 ^g^
∑ Trim *	32.95 ± 0.25 ^a^	32.87 ± 0.33 ^a^	32.17 ± 0.26 ^b^	31.20 ± 0.28 ^c^	30.86 ± 0.13 ^c^	32.59 ± 0.07 ^ab^	32.40 ± 0.20 ^ab^	26.58 ± 0.37 ^d^	25.62 ± 0.24 ^e^
mDP	3.8 ± 0.1 ^ab^	3.8 ± 0.1 ^ab^	3.7 ± 0.0 ^ab^	3.6 ± 0.0 ^a^	3.7 ± 0.1 ^ab^	3.7 ± 0.0 ^ab^	3.7 ± 0.1 ^ab^	3.8 ± 0.0 ^ab^	3.9 ± 0.0 ^b^
%G	6.5 ± 0.1 ^a^	6.4 ± 0.0 ^ab^	6.2 ± 0.1 ^ab^	5.9 ± 0.1 ^b^	6.1 ± 0.1 ^b^	6.2 ± 0.1 ^ab^	6.3 ± 0.3 ^ab^	6.2 ± 0.3 ^ab^	6.4 ± 0.1 ^ab^
%P	32.0 ± 0.8 ^a^	31.3 ± 0.8 ^a^	31.3 ± 0.3 ^a^	30.0 ± 0.0 ^a^	31.1 ± 1.4 ^a^	31.9 ± 1.2 ^a^	31.1 ± 0.5 ^a^	30.3 ± 0.5 ^a^	30.9 ± 0.6 ^a^

Data are presented as average value ± standard deviation in mg/L * and g/L ** over three analytical replicates (*n* = 3). ANOVA to compare data; different letters in each row indicate statistical difference between wines (Tukey’s test, *p* < 0.05). Abbreviations: TP, total phenolics; TT, total tannins; (+)-C, catechin; (−)-EC, epicatehin; B1, B2, B3,B4,C1 and T2-procyanidins B1, B2, B3, B4, C1 and T2; ∑ Mon, sum of flavan-3-ol monomers; ∑ Dim, sum of flavan-3-ol dimers; ∑ Trim, sum of flavan-3-ol trimers; mDP, mean degree of polymerization; %G, percentage of galloylation; %P, percentage of prodelphinidins.

**Table 3:** Effect of partial dealcoholization operating parameters on the anthocyanins concentrations and chromatic characteristics in Plavac mali wine.

Operating Parameters									
	Control	Dealcoholized Wine							
Membrane	/	ACM3	ACM3	TS80	TS80	ACM3	ACM3	TS80	TS80
TMP (bar)	/	25	35	25	35	25	35	25	35
Process	/	−2%	−2%	−2%	−2%	−4%	−4%	−4%	−4%
Analytical parameters									
TA *	248.6 ± 2.5 ^a^	247.4 ± 6.9 ^a^	236.7 ± 6.1 ^ab^	234.4 ± 6.0 ^ab^	229.3 ± 7.5 ^ab^	243.3 ± 10.6 ^ab^	241.6 ± 9.5 ^ab^	185.5 ± 7.9 ^c^	180.4 ± 3.0 ^c^
Df *	21.31 ± 0.02 ^a^	21.29 ± 0.02 ^a^	21.19 ± 0.15 ^a^	21.37 ± 0.07 ^a^	21.27 ± 0.05 ^a^	21.26 ± 0.18 ^a^	21.19 ± 0.14 ^a^	20.19 ± 0.11 ^a^	20.10 ± 0.07 ^a^
Cy *	18.61 ± 0.01 ^a^	18.62 ± 0.09 ^a^	18.61 ± 0.04 ^a^	18.58 ± 0.00 ^b^	18.58 ± 0.00 ^b^	18.61 ± 0.01 ^a^	18.60 ± 0.02 ^a^	18.58 ± 0.00 ^b^	20.39 ± 0.18 ^b^
Pt *	22.27 ± 0.04 ^a^	22.18 ± 0.03 ^a^	22.21 ± 0.06 ^a^	22.28 ± 0.23 ^a^	22.30 ± 0.10 ^a^	22.15 ± 0.20 ^a^	22.10 ± 0.18 ^a^	20.57 ± 0.12 ^b^	22.39 ± 0.18 ^b^
Pn *	20.84 ± 0.05 ^ab^	20.90 ± 0.06 ^a^	20.71 ± 0.14 ^ab^	20.73 ± 0.04 ^ab^	20.69 ± 0.15 ^ab^	20.86 ± 0.11 ^ab^	20.63 ± 0.13 ^b^	19.86 ± 0.05 ^c^	19.72 ± 0.09 ^c^
Mv *	59.99 ± 0.15 ^a^	59.35 ± 0.78 ^a^	57.46 ± 1.78 ^a^	59.32 ± 0.58 ^a^	58.17 ± 2.81 ^a^	60.10 ± 0.24 ^a^	56.85 ± 3.30 ^a^	44.02 ± 0.74 ^b^	42.36 ± 0.48 ^b^
PnAc *	18.99 ± 0.04 ^a^	18.99 ± 0.05 ^a^	18.94 ± 0.03 ^a^	18.94 ± 0.05 ^a^	18.95 ± 0.04 ^a^	18.98 ± 0.05 ^a^	18.96 ± 0.03 ^a^	18.72 ± 0.07 ^b^	18.68 ± 0.06 ^b^
MvAc *	26.29 ± 0.10 ^a^	26.34 ± 0.04 ^a^	25.94 ± 0.36 ^a^	26.18 ± 0.16 ^a^	26.00 ± 0.54 ^a^	26.48 ± 0.24 ^a^	25.80 ± 0.20 ^a^	23.28 ± 0.08 ^b^	22.94 ± 0.10 ^b^
PnCm *	18.95 ± 0.01 ^a^	18.96 ± 0.01 ^a^	18.93 ± 0.03 ^a^	18.95 ± 0.01 ^a^	18.94 ± 0.03 ^a^	18.96 ± 0.00 ^a^	18.93 ± 0.03 ^a^	18.71 ± 0.00 ^b^	18.68 ± 0.00 ^b^
MvCm *	23.08 ± 0.02 ^a^	22.70 ± 0.14 ^ab^	22.80 ± 0.14 ^ab^	23.18 ± 0.03 ^a^	23.10 ± 0.05 ^a^	23.01 ± 0.05 ^a^	22.18 ± 0.17 ^ab^	20.91 ± 0.05 ^c^	20.60 ± 0.11 ^c^
∑ AcyGlc *	143.02 ± 0.18 ^a^	142.33 ± 0.86 ^a^	140.17 ± 2.11 ^a^	142.28 ± 0.46 ^a^	141.01 ± 3.02 ^a^	142.97 ± 0.06 ^a^	139.38 ± 3.74 ^a^	123.21 ± 0.95 ^b^	121.14 ± 0.46 ^b^
∑ AcyAc *	45.28 ± 0.02 ^ab^	45.33 ± 0.03 ^ab^	44.89 ± 0.38 ^ab^	45.12 ± 0.17 ^ab^	44.95 ± 0.55 ^ab^	45.46 ± 0.26 ^ab^	44.77 ± 0.22 ^b^	42.00 ± 0.08 ^c^	41.63 ± 0.10 ^cd^
∑ AcyCm *	42.03 ± 0.02 ^a^	41.65 ± 0.14 ^c^	41.73 ± 0.16 ^bc^	42.13 ± 0.04 ^a^	41.94 ± 0.06 ^ab^	42.06 ± 0.06 ^a^	41.12 ± 0.18 ^d^	39.62 ± 0.05 ^e^	39.29 ± 0.11 ^f^
L	71.94 ± 1.01 ^cd^	71.64 ± 0.51 ^cde^	74.37 ± 1.32 ^ab^	74.91 ± 1.81 ^ab^	75.94 ± 1.05 ^a^	70.87 ± 0.38 ^de^	73.32 ± 0.38 ^bc^	71.23 ± 1.16 ^cde^	69.43 ± 0.08 ^e^
a	25.18 ± 0.96 ^ab^	25.30 ± 0.80 ^ab^	22.29 ± 1.37 ^cd^	21.74 ± 1.81 ^cd^	20.67 ± 1.09 ^d^	24.92 ± 0.20 ^ab^	23.36 ± 0.48 ^b^	24.74 ± 0.98 ^cd^	26.33 ± 0.11 ^a^
b	5.60 ± 0.13 ^cd^	5.34 ± 0.56 ^d^	6.00 ± 0.13 ^bc^	6.02 ± 0.06 ^bc^	6.38 ± 0.05 ^ab^	6.80 ± 0.07 ^a^	6.50 ± 0.22 ^ab^	6.90 ± 0.10 ^a^	6.47 ± 0.20 ^ab^
C_ab_	25.81 ± 0.92 ^ab^	25.87 ± 0.68 ^ab^	23.09 ± 1.35 ^cd^	22.56 ± 1.74 ^cd^	21.64 ± 1.05 ^d^	25.83 ± 0.21 ^ab^	24.25 ± 0.52 ^b^	25.71 ± 0.97 ^ab^	27.11 ± 0.13 ^a^
h	0.22 ± 0.01 ^c^	0.22 ± 0.03 ^c^	0.26 ± 0.01 ^b^	0.27 ± 0.02 ^ab^	0.30 ± 0.01 ^a^	0.27 ± 0.01 ^ab^	0.27 ± 0.01 ^ab^	0.28 ± 0.01 ^ab^	0.24 ± 0.01 ^bc^

Data are presented as average value ± standard deviation in mg/L * over three analytical replicates (*n* = 3). ANOVA to compare data; different letters in each row indicate statistical difference between wines (Tukey’s test, *p* < 0.05). Abbreviations: TA, total anthocyanins; Df, delphinidin-3-*O*-glucoside; Cy, cyanidin-3-*O*-glucoside; Pt, petunidin-3-*O*-glucoside; Pn, peonidin-3-*O*-glucoside; Mv, malvidin-3-*O*-glucoside; PnAc, peonidin-3-*O*-(6-*O*-acetyl)glucoside; MvAc, malvidin-3-*O*-(6-*O*-acetyl)glucoside; PnCm, Peonidin-3-*O*-(6-*O*-p-coumaroyl))glucoside; MvCm, malvidin-3-*O*-(6-*O*-*p*-coumaroyl)glucoside; ∑ AcyGlc, sum of anthocyanin glucosides; ∑ AcyAc, sum of anthocyanin acetylglucosides; ∑ AcyCm; sum of anthocyanin coumaroylglucosides.

## Data Availability

The original contributions presented in the study are included in the article; further inquiries can be directed to the corresponding author.
